# Brain–Periphery Interactions in Huntington’s Disease: Mediators and Lifestyle Interventions

**DOI:** 10.3390/ijms25094696

**Published:** 2024-04-25

**Authors:** Johannes Burtscher, Barbara Strasser, Giuseppe Pepe, Martin Burtscher, Martin Kopp, Alba Di Pardo, Vittorio Maglione, Andy V. Khamoui

**Affiliations:** 1 Institute of Sport Sciences, University of Lausanne, 1015 Lausanne, Switzerland; 2 Ludwig Boltzmann Institute for Rehabilitation Research, 1100 Vienna, Austria; barbara.strasser@med.sfu.ac.at; 3 Faculty of Medicine, Sigmund Freud Private University, 1020 Vienna, Austria; 4 IRCCS Neuromed, 86077 Pozzilli, Italy; giuseppe.pepe@neuromed.it (G.P.); dipardoa@hotmail.com (A.D.P.); vittorio.maglione@neuromed.it (V.M.); 5 Department of Sport Science, University of Innsbruck, 6020 Innsbruck, Austria; martin.burtscher@uibk.ac.at (M.B.); martin.kopp@uibk.ac.at (M.K.); 6 Department of Exercise Science and Health Promotion, Florida Atlantic University, Boca Raton, FL 33458, USA; akhamoui@fau.edu; 7 Institute for Human Health and Disease Intervention, Florida Atlantic University, Jupiter, FL 33458, USA

**Keywords:** Huntington’s disease, neurodegeneration, gut–brain, muscle–brain, inter-organ signaling, circulating messengers, exercise, diet

## Abstract

Prominent pathological features of Huntington’s disease (HD) are aggregations of mutated Huntingtin protein (mHtt) in the brain and neurodegeneration, which causes characteristic motor (such as chorea and dystonia) and non-motor symptoms. However, the numerous systemic and peripheral deficits in HD have gained increasing attention recently, since those factors likely modulate disease progression, including brain pathology. While whole-body metabolic abnormalities and organ-specific pathologies in HD have been relatively well described, the potential mediators of compromised inter-organ communication in HD have been insufficiently characterized. Therefore, we applied an exploratory literature search to identify such mediators. Unsurprisingly, dysregulation of inflammatory factors, circulating mHtt, and many other messenger molecules (hormones, lipids, RNAs) were found that suggest impaired inter-organ communication, including of the gut–brain and muscle–brain axis. Based on these findings, we aimed to assess the risks and potentials of lifestyle interventions that are thought to improve communication across these axes: dietary strategies and exercise. We conclude that appropriate lifestyle interventions have great potential to reduce symptoms and potentially modify disease progression (possibly via improving inter-organ signaling) in HD. However, impaired systemic metabolism and peripheral symptoms warrant particular care in the design of dietary and exercise programs for people with HD.

## 1. Introduction

The brain is in constant interaction with peripheral organs. Deficits in this communication are thought to be involved in a multitude of diseases, including neurodegenerative diseases. This possibility currently is avidly explored for the gut–brain axis [1], but increasingly also for the interaction of the brain with other organs and tissues, including the heart [2] and skeletal muscle [3]. Here, we aim to explore the current knowledge on mediators of deficits in brain–periphery interactions in Huntington’s disease (HD), a neurodegenerative disease, in which brain pathology is paralleled by substantial peripheral abnormalities [4,5].

The autosomal dominant disorder Huntington’s disease is caused by the multiplication of a CAG-repeat in exon 1 of the *Huntingtin* (*Htt*) gene [6], resulting in a prolonged poly-glutamine stretch in Huntingtin protein (Htt), a large protein, expressed in almost all human and mouse tissues [7]. While repeats of 36–39 CAGs are associated with reduced penetrance, more than 39 repeats are considered fully penetrant [8]. HD is characterized by neurodegeneration, positive and negative motor symptoms, including chorea (involuntary jerky movements), dystonia (uncontrolled muscle contractions), impaired motor and balance control, and hypokinetic motor symptoms, especially in later disease stages [9,10]. In addition, non-motor symptoms, such as cognitive impairment (especially of executive functions and motor skill learning) and psychiatric symptoms are frequent [10]. Despite the clear genetic cause of HD, disease-modifying treatments are lacking, due to an incomplete understanding of the physiological functions of Htt and complex interactions between pathological processes in different organs. Htt plays roles in the cellular regulation of transcription, cellular morphology and cytoskeleton functions, metabolism, synapses, and apoptosis [7,11]. Moreover, Htt is involved in systemic processes, such as immune system function, for example, by the regulation of macrophage physiology [12].

In neurons, mutated Htt (mHtt, Htt with polyglutamine expansion) forms intracellular nuclear inclusions and cytosolic aggregates [13,14], impairing different cellular functions, such as transcription [15] and energy production, resulting in oxidative damage and ultimately in the death of vulnerable cells, in particular of medium spiny GABAergic neurons in the dorsal striatum (caudate nucleus and putamen), leading to atrophy in the affected brain regions (primarily striatum, cortical regions, substantia nigra, and others) [10]. Other neuropathological features of HD besides mHtt toxicity include reduced brain-derived neurotrophic factor (BDNF) levels [16]; neuroinflammation, mitochondrial dysfunction, and oxidative stress [17]; impaired cellular signaling [18]; cerebrovascular deficits [19,20], as well as impaired cerebrospinal fluid flow and compromised clearance of toxic material from the brain [21]. The different aspects of HD pathology also result in changes in regional blood flow and the activity of numerous brain regions, including hypoperfusion and hypometabolism of the basal ganglia and cortical regions, as well as the downregulated activity of cortical components of the executive networks, demonstrated extensively in mouse models of HD and people with HD [22,23,24,25,26,27,28,29].

Aside from brain deficits, HD is associated with various systemic deficits [4,5], comprising metabolic impairment in multiple organs, atrophy of skeletal muscle, cardiovascular problems, increased inflammation [30], and generally disrupted organismal homeostasis, reflected for example by disrupted circadian rhythms [31]. HD-related metabolic vulnerabilities outside the nervous system are likely important players in HD progression and potentially are also involved in the generation of brain-related symptoms [4,7]. The disruption of the physiological interactions between peripheral organs and the brain may compromise organismal homeostasis and adequate systemic stress responses (to mHtt toxicity or different stressors) in HD and drive disease progression.

Taken together, metabolic alterations in the brain and peripheral organs are determinants of disease progression in Huntington’s disease (HD) and other neurodegenerative diseases [32], and a detailed understanding of these deficits is potentially important for prognosis and the development of novel strategies to slow down or modify disease progression. The progressing complex systemic deficits characterizing HD likely are also one reason why targeted pharmacological treatment approaches until today are not efficient in HD patients. Here, we aim to explore possible mediators of brain–periphery interactions that have been reported in the literature and put them in the context of relevant peripheral deficits in HD. In addition, we evaluate the risks and benefits of different healthy lifestyles that can modulate peripheral metabolic deficits, brain–periphery communication, and/or brain functions, either directly or indirectly. We attempt to derive preliminary conclusions for beneficial lifestyles in HD and suggest questions that should be explored in future research.

### 1.1. Systemic Metabolic Vulnerabilities in Huntington’s Disease

Different HD-related metabolic impairments in various organs likely inter-dependently influence disease progression. In combination with metabolic alterations and impaired inter-tissue crosstalk, structural deterioration in tissues such as skeletal muscle, heart, adipose tissue, and bone leads to cachexia and impaired gut/gut-microbiome structure and function may influence systemic metabolism, including of the brain [4].

It has been speculated that a brain energy deficit in HD is linked to alterations in systemic metabolic processes [33]. People with HD indeed usually experience CAG-repeat-dependent weight loss [34], a reduced body mass index (BMI), and lower lean mass [35], despite increased appetite and calorie intake [36]. This has initially been linked to a reported increased resting energy expenditure in HD [37], although the authors of this study observed no differences in overall energy expenditure levels as compared to controls, at least partly due to lower voluntary physical activity levels in HD patients. Paradoxically, higher food intake and appetite are common already in early disease stages and later [36,38,39]. The confirmation of higher calculated energy uptake together with reduced BMI also in presymptomatic HD mutation carriers as compared to non-carrier controls suggests that these metabolic effects are not merely due to increased energy expenditure of patients due to involuntary movements [38]. A subsequent study found a similar fat-free mass and basic metabolic rates in people with HD as compared to healthy controls despite reduced BMI [40]. Body weight has been identified as a predictor for the rate of HD progression, with higher BMI being associated with slower functional decline in an observational study [41]. Moreover, the reported reduced bone mineral density for people with HD, even in the pre-manifest stage [42,43], which is similar to observations in the R6/2 HD-mouse model [44], suggests early changes in body composition that may contribute to disease progression.

The basis of the alterations in body composition in HD are likely impaired metabolic pathways, which can be observed in HD brain and other organs. Dysregulated glucose homeostasis and impaired insulin responses in HD patients, also if they were not diabetic [45], and decreased availabilities of the branched amino acids valine, leucine, and isoleucine that are associated with weight loss in (human) HD [46], indicate deficits in glucose metabolism and Krebs cycle activity. In addition, alterations in lipid metabolism are characteristic of human and model HD and include, for example, cholesterol [47] and sphingolipid metabolism [48].

HD-related abnormalities in organs like liver, pancreas, and adipose tissue (for a review see [4]), as well as in the gut and skeletal muscle (discussed in more detail in the next chapter) are likely all importantly involved in systemic metabolic deficits in HD and consequences on brain, body composition and the brain–periphery communication.

### 1.2. Mouse Models of Huntington’s Disease to Study Brain–Periphery Interactions

Different animal models of HD have been used to study crosstalk between brain and peripheral tissues. Among them are parabiosis models, in which the circulatory systems of two mice (e.g., an HD mouse with a wild-type mouse) are surgically connected. If the involved mice are of different ages, the model is called heterochronic parabiosis.

HD mouse models can be categorized as transgenic or knock-in models. In transgenic models, a human *Htt* gene with a high CAG-repeat number has been inserted in the mouse genome. The inserted *Htt* can thereby be full-length (e.g., in BACHD, YAC) or partial (e.g., R6/2, N171-82Q). Knock-in HD models are characterized by either simply expanded CAG repeats in mouse Htt (e.g., hdh (CAG150)) or a chimeric murine/human expanded CAG repeat sequence in the mouse endogenous *Htt*-homologous gene (e.g., CAG140, zQ175).

Transgenic R6/2 mice (expressing an expanded CAG repeat—about 150—stretch of the human *Htt* exon 1) are models of an aggressive disease progression, with death of the animals occurring at 12–15 weeks [49]. In other HD mouse models, like CAG140 or zQ175 mice, the disease progresses less rapidly: CAG140 or zQ175 mice live about 1.5 to 2 years [50]. Similarly, mice expressing human full-length mHtt, like the yeast artificial chromosome 128 (YAC128) model or bacterial artificial chromosome-mediated transgenic HD (BACHD) mice, develop HD-like pathology more gradually [51].

## 2. Potential Mediators of Brain–Periphery Communication

While many peripheral deficits in HD are relatively well characterized [4], their interaction with the brain and brain pathology is only just beginning to be investigated. Different organs interact among themselves via various means and these interactions are likely of great relevance for the development of chronic diseases and for aging in general [52]. However, the communications channels that are important or impaired in HD are not well characterized [4]. Therefore, we performed a literature search aiming to identify research investigating brain–periphery interaction in HD and to evaluate, which modes of inter-organ communication may be the most relevant in HD and potentially have not been sufficiently investigated.

We applied an explorative search strategy on PubMed using the following keyword search: “Huntington’s disease” AND (circulating OR brain - periphery OR mitokines OR exerkines OR gut - brain OR liver-brain OR muscle - brain OR heart - brain). The search yielded 177 results and relevant original research publications were extracted (publications reporting peripheral deficits without investigating potential mechanisms for inter-organ communication). The identified reviews were also screened for references to additional original research publications on inter-organ communication and relevant hits were included in our results. The results are summarized in the following Sections.

### 2.1. Circulating mHtt, Inflammatory, and Immune Factors

The applied search strategy yielded several related to mHtt, inflammatory factors, and dysregulation of the immune system, which are summarized in Table 1.

#### 2.1.1. mHtt

Htt—or mHtt—is expressed ubiquitously [62] and likely contributes to systemic deterioration by being released into the circulation, probably facilitated by (cerebro-)vascular deficits and a compromised blood–brain barrier [4]. Mounting evidence suggests that mHtt can be transferred between neurons, as shown in the occurrence of mHtt aggregation in fetal grafts that had been transplanted in HD brains [63], thereby possibly contributing to pathology spreading. But mHtt may also be transferred between different cell types and tissues, and even over long distances between organs. In rodent models, intra-ventricular implantation of HD-patient derived mHtt-expressing fibroblasts or induced pluripotent stem cells led to the—likely exosome-mediated—transmission of mHtt pathology to host cells and resulted in neuroinflammation, gliosis, and striatal cell loss, as well as motor and cognitive symptoms resembling HD symptomatology [64]. Intracerebral injection of homogenates from HD brain aggravated HD-like symptoms in HD-model mice (BACHD) and led to the occurrence of mHtt pathology in wild-type mice or non-human primates, however, without inducing behavioral symptoms [65]. In contrast, the intraventricular injection of exogenous, recombinant human fibrillar mHTT (Q48) Exon1 induced cognitive and anxiety-like symptoms in wild-type mice and exacerbated those symptoms in HD-model mice (R6/2), although the mHtt fibrils were not observed any more 14 months after injection [66]. Importantly, in this study, peripheral application of mHtt fibrils triggered an immune response but did not result in behavioral alterations [66]. Other studies suggest that mHtt aggregates can induce the formation of mHtt pathology in nearby cells or even distant tissues. Rieux and colleagues [53] used a mouse parabiosis approach, in which the circulatory systems of zQ175 HD model mice were joined with those of wild-type mice. This resulted in the deposition of mHtt in multiple organs and led to organ-specific alterations of mitochondrial protein levels in the wild-type mice. A recent study found that mHtt can be actively transported from motor neurons to muscle cells in iPSC models, causing molecular and functional deficits in the receiving cells [67].

If and how mHtt-related pathology is propagated between cells and tissues in human HD remains a topic of debate, as discussed in recent reviews [68,69].

#### 2.1.2. Immune System Dysregulation and Inflammation

Inflammation is strongly linked to neurodegenerative diseases in general and to HD specifically [30,70]. In addition, the contribution of inflammation in peripheral tissues to HD progression is increasingly considered and the related evidence has been recently summarized [71].

Our search strategy led to the identification of only a few studies related to circulating inflammatory markers and the references in Table 1 are a far from complete overview of such factors in HD. A recent systematic review and meta-analysis summarized changes in inflammatory markers in more detail [72].

Cellular injuries, e.g., of skeletal muscle, are tightly connected to inflammatory responses and are discussed in the next Section.

### 2.2. Circulating Metabolites, Muscle Injury Markers

Brain and systemic metabolic abnormalities are common in HD. It is thus not surprising that the levels of several metabolites are altered in the blood of people with HD or in animal models of HD (Table 2).

#### 2.2.1. The Muscle–Brain Axis

The results in Table 2 suggest alterations in circulating factors indicating abnormalities in skeletal muscle or generally energy metabolism in HD [74,75] or mouse models of HD [73]. Muscle metabolism is well known to be impaired in HD [4,30,78,79]. The deficits include mitochondrial dysfunction and reduced ATP production (assessed from phosphocreatine recovery), even in presymptomatic HD-mutation carriers [30,80]. Accordingly, increased plasma levels of lactate in symptomatic HD patients and a reduced anaerobic threshold in both symptomatic and presymptomatic carriers of the HD mutation have been reported during exercising [75]. In various rodent HD models, structural changes in skeletal muscles have been characterized and include changes in myofiber atrophy, type switching, and denervation (for reviews see [4]). In addition, mHtt aggregates have been observed in muscle fibers in HD models and people with HD [30].

Impaired skeletal muscle metabolism in HD leads to muscle wasting, as recently reviewed [81], and is probably the main factor in body weight loss in HD [4]. Cachexia and muscle wasting are detrimental manifestations in many neurodegenerative diseases—for example, predicting survival in Alzheimer’s disease patients [82]—but they are especially major components of HD [79]. Muscle wasting also characterizes major HD mouse models, such as R6/2 and HdhQ150, where measurable loss of muscle mass was found in all major skeletal muscle types [83]. Muscles of R6/2 mice further have been shown to be more vulnerable to calcium-induced stress [84], indicating impaired stress responses in HD muscles. Interestingly, the expression of mHtt (Q72) in neurons of flies (Drosophila melanogaster) also affected muscle performance in climbing and flying, suggesting negative consequences of brain pathology on skeletal muscle [85].

Based on the possibility of impaired brain–muscle crosstalk in HD, van Diemen and colleagues [86] hypothesized that brain (visual cortex) mitochondrial function was correlated with peripheral mitochondrial functions (calf muscle in vivo, circulating peripheral blood mononuclear cells ex vivo) in people with manifest HD (39–60 CAG repeats, no healthy controls). Such a correlation could suggest inter-organ mitochondrial crosstalk involved in signaling between the brain and muscles [87] and could be an important aspect of impaired brain–periphery communication in HD. However, when assessing mitochondrial function in vivo by phosphorous magnetic resonance spectroscopy, they did not find such a correlation, and only brain mitochondrial function was correlated with the Unified Huntington disease rating score (UHDRS) total motor score [86]. Importantly, this study did not include a control group. It thus remains to be determined if the correlations of central and peripheral mitochondria are different in people with HD and if mitochondrial inter-organ signaling may be specifically impaired in HD, although another study also reports a lack of correlation between platelet mitochondrial complex I and I + III activities and the cerebral metabolic rate of oxygen in people with HD, Parkinson’s disease, or without neurological diseases [88]. Van Diemen et al. provided a mitochondrial function booster (three doses of SBT-020) in a randomized, double-blinded, placebo-controlled trial to people with manifest HD but did not observe significant improvements in mitochondrial functions in calf muscle, peripheral blood mononuclear cells, or visual cortex [89]. They suggested that SBT-020 may be more efficient in people with more pronounced mitochondrial dysfunction because they observed better effects when mitochondrial impairment was greater.

Overall, metabolic alterations in HD muscles lead to reduced muscle function and exercise capacity. This results in a situation in which exercise limitations and disease-related metabolic deficits reciprocally lead to a worsening of muscle metabolism efficiency. Since active muscles communicate with the brain via various mechanisms, including myokine-signaling (molecules released from muscle and exerting effects on cells/tissues), direct adverse effects on the brain are to be expected [90]. A few studies indirectly investigated the potential benefits of the exogenous administration of myokines in cell and animal models of HD. Mimicking brain-derived neurotrophic factor (BDNF) action using small molecules (7,8-dihydroxyflavone and 4′-dimethylamino-7,8-dihydroxyflavone) in N171-82Q for example reduced motor deficits and brain atrophy, at least partially by mitigating impaired neurogenesis [91]. BDNF is increased in the brain after exercise and it has been shown to be expressed and released from skeletal muscle, thus potentially directly acting on the brain, since it can penetrate the blood–brain barrier [92]. However, specific studies on muscle–brain communication in HD and the role of myokines and exerkines in HD disease progression are lacking.

#### 2.2.2. The Gut–Brain Axis

The results in Table 2 also indicate alterations in gut–brain communication in an HD mouse model [76,77]. Kong and colleagues performed shot-gun sequencing and blood metabolomics in the R6/1 HD mouse model to characterize their gut microbiome composition longitudinally throughout disease progression [76]. These authors report early signs of a potentially compromised gut microbiome in the pre-motor symptomatic stage and dysbiosis and compromised gut microbiome function during the late symptomatic stage. They suspect that impaired microbiome function in HD model mice resulted in compromised gut–brain interaction via changes in the circulating metabolome, specifically related to butanoate metabolism [76], in which short-chain fatty acids and short-chain alcohols primarily generated by fermentation in the gut are processed.

Increasing evidence indicates a disrupted gut–brain axis in models of, and people with, neurodegenerative diseases, including HD [93,94,95,96,97].

Using fecal samples from human HD gene carriers, a different gut microbiome composition than in healthy controls has been shown and this was correlated with cognitive and clinical assessments [98]. This finding confirmed previous results from mouse models and demonstrated some degree of translational validity. In the R6/2 model, elevated intestinal permeability and a shifted proportion of the dominant gut-resident phyla (with the composition changing towards reduced relative Firmicutes and higher Bacteroidetes abundance) have been found and linked to reductions both in body length and colon length [99]. Fecal microbiota transplants from wild-type into R6/1 mice improved cognition, especially in females [94].

Enteric cells in the gut have also been found to contain mHtt aggregates in mouse models of HD and people with HD [100]. In addition, impaired nutrient absorption in the gastrointestinal tract has been reported and is potentially linked to unintentional weight loss in HD [5].

The alteration of the gut microbiome may also affect mHtt pathology. In flies (Drosophila melanogaster), targeting gut bacteria by antibiotics reduced mHtt aggregation and motor defects [101]. Similarly, artificial colonization of HD flies with E. coli aggravated mHtt aggregation and motor defects and reduced lifespan [101].

### 2.3. Circulating Lipid and Peptide Messengers, Hormones

The levels of various lipid and peptide messengers and of several hormones regulating, for example, appetite, systemic metabolism, or psychological stress, are known to be changed in HD, and related publications are summarized in Table 3. The different clusters of results are discussed in the following Sections.

#### 2.3.1. Lipids

As a major membrane and myelin sheath component, cholesterol is essential for brain homeostasis. Cholesterol itself does not cross the blood–brain barrier and has to be synthesized in the brain (in the adult brain mainly by astrocytes) in an energy and oxygen-dependent manner [47]. Cholesterol metabolism is compromised in HD brain, and Kacher and colleagues HD [115] demonstrated in zQ175 HD mice that upregulating cholesterol 24-hydrolase (CYP46A1), the rate-limiting enzyme for cholesterol degradation in the brain, via gene therapy, could restore cholesterol homeostasis, providing neuroprotection.

While deregulated total cholesterol levels have been reported in people with pre-manifest and manifest HD in blood [103], other studies did not observe significant changes in circulating cholesterol but rather in its catabolites. Reduced levels of the cholesterol catabolite 24S-hydroxycholesterol (24S-OHC) have been observed in Q175 mouse blood [108] and in the plasma of HD patients [104,105]. These findings are thought to reflect changes in cholesterol metabolism in the brain [108] and since they correlate with clinical scores [104,105], they may be informative biomarkers (see Table 3). How changes in brain cholesterol-related metabolites might affect peripheral organs remains to be investigated.

In humans, the use of 3-hydroxy-3-methyl-glutaryl-coenzyme A reductase inhibitors (statins), a widely prescribed class of cholesterol-reducing drugs, has been shown to be associated with a delayed onset of HD [116]. However, these beneficial effects may primarily be due to the anti-oxidative effects of statins. It has been pointed out in response to the publication that BMI may be a confounding factor in this study [117]. In addition, statins exert anti-inflammatory effects and promote autophagy [118], which could all be beneficial in HD. Therefore, it is currently unclear whether the direct modulation of circulating cholesterol or its metabolites is beneficial in HD.

The plasma levels of other lipid messengers that have been suggested to play a role in HD, such as the cannabinoids anandamide and 2-arachidonoylglycerol, were not changed in presymptomatic and symptomatic HD in a recent study, and their levels were also not correlated with clinical scores [102].

#### 2.3.2. Hypothalamic Hormones Regulating Systemic Metabolism

The hypothalamus is a key coordinator of behavioral responses based on metabolic status, which the hypothalamus senses via numerous peripheral cues, including insulin, ghrelin, and leptin, as reviewed elsewhere [32]. While the release of the pancreatic hormone insulin depends on blood glucose levels, the elevated release of leptin by adipocytes induces satiety and the gastrointestinal-tract-derived ghrelin stimulates feeding. Ghrelin and leptin are key neuropeptide hormones regulating the crosstalk between systemic metabolism and damage in the brain [119]. Several preclinical studies in HD mouse models confirm dysregulated systemic metabolism and associated changes in glucose and insulin homeostasis, which were described in people with HD (Lalić et al., 2008 [45]), by demonstrating increased circulating levels of these molecules [44,111,112], as summarized in Table 3. Another study in people with HD, however, did not find changes in circulating glucose or insulin levels in either plasma or cerebrospinal fluid [106]. However, this study found higher ghrelin and lower leptin levels in the plasma of individuals with HD (but not in cerebrospinal fluid), indicating a negative energy balance.

In addition, low plasma leptin levels indicate reduced adipose tissue mass [120], and, accordingly, HD patients (especially male patients) have reduced body fat [43]. In mouse HD models (R6/2 and CAG 140 knock-in) reduced circulating leptin levels have also been found and in combination with alterations in adipose tissue indicate HD-related deficits in lipid accumulation in adipocytes and adipocyte signaling [113].

#### 2.3.3. Stress Hormones

The hypothalamus is also a key element in the systemic stress response and via the hypothalamic–pituitary–adrenal axis regulates glucocorticoid release and thereby the human stress hormone cortisol or its rodent homolog corticosterone. High circulating cortisol/corticosterone has been found both in human HD [44] and in mouse models thereof [44,109,110]. Besides their role in the regulation of (psychological) stress responses, glucocorticoids are also known regulators of muscle mass [121] and, thus, the hypothalamus is also involved in muscle phenotypes in HD [4,44], thereby potentially playing a role in the regulation of the muscle–brain axis. Although the role of the hypothalamus in HD progression and especially in HD-associated peripheral symptoms requires more investigation, an important involvement of hypothalamic deficits in the metabolic impairments of HD patients is likely [32].

Besides the messenger molecules discussed in this Section, different types of RNAs are recognized as communication mediators between tissues.

### 2.4. RNAs and Extracellular Vesicles

There is substantial and growing interest in the involvement of different types of RNAs, e.g., long non-coding RNAs or microRNAs, in the pathogenesis of neurodegenerative diseases and their potential as biomarkers [122,123,124,125]. Unsurprisingly, also in HD, the levels of circulating RNAs have been reported to be significantly dysregulated [126]. Circulating RNAs, like many other molecules, can be transported in extracellular vesicles, or exosomes (a type of extracellular vesicles secreted from cells and originating from intracellular multivesicular bodies that fuse with the plasma membrane). The role of extracellular vesicles in neurodegenerative diseases is not fully elucidated but likely is importantly involved in inter-organ communication and the interdependence of deficits/pathology in the brain and the periphery [127].

Table 4 summarizes the results related to RNAs and extracellular vesicles identified by our literature search on potential mediators of brain–periphery interactions.

Various RNAs have been suggested as potential biomarkers of HD, based on altered levels compared to healthy controls [130,131]. Interestingly, customized dietary interventions improved microRNA dysregulations in people with HD, which was paralleled by reduced unintentional weight loss and increased fat mass and circulating leptin levels [132].

In a model of heterochronic parabiosis, in which the circulatory systems of young wild-type mice were connected with those of older HD mice, improved molecular markers (mitochondrial biogenesis, cell death), reduced weight loss, and increased cognitive function and survival in the HD model mice [133]. The authors of that study demonstrated in vitro (on cultures from R6/2 neural stem cells) that exosomes from young mouse blood serum seem to partially mediate these effects. Recently, studies by Neueder and colleagues [128,129] suggested that extracellular vesicles are also involved in human HD pathology progression. While most general characteristics of the extracellular vesicles were not changed in the blood of people with HD, increased populations of smaller extracellular vesicles and differences in RNA content were observed [129] that point to the liver as the main organ involved in releasing HD-specific extracellular vesicles.

### 2.5. Cardio- and Neurovascular Changes

HD patients commonly suffer from autonomic nervous system dysfunction (dysautonomia) [134], associated other cardiovascular risk factors [135], and impaired heart muscle function [136], symptoms that have also been reproduced in mouse models of HD [137,138,139]. Accordingly, cardiovascular events represent important risk factors for premature mortality in HD [134]. The reasons for increased dysautonomia and cardiovascular abnormalities are not fully understood but are likely due to an impaired interconnectivity between the cardiovascular and nervous systems [2]. Impaired regulation of peripheral vasodilation by nitric oxide has been shown in R6/2 mice [140] and dietary modulation of nitric oxide levels has been demonstrated in R6/1 mice to influence disease progression and symptoms [141] (see Table 5).

In addition, the neurovascular system is frequently impaired in neurodegenerative diseases, including HD [144]. The neurovascular system orchestrates cerebral blood flow and the exchange of molecules between the brain and the circulation (blood–brain barrier). Changes in blood flow and permeability influence brain–periphery communication and dysfunction can lead to nutrient (including glucose) and oxygen (and thereby energy) depletion, reduced regulation of influx of circulating substances (e.g., inflammatory molecules), or impaired cerebral waste clearance, all central factors in neurodegenerative diseases. The literature summarized in Table 5 demonstrates regional changes in cerebral blood flow [23,24,28,142,143] and suggests impaired blood–brain barrier function [19] in people with HD. The changes in cerebral blood flow particularly affect the striatum in HD, leading to reduced perfusion in this vulnerable network.

Overall, impairments in the cardiovascular and neurovascular system can result in regional hypometabolism and likely contribute to a dysregulation of brain–periphery communication, both by enhancing the permeability of the brain to harmful circulating substances and by reducing not only access of affected tissues to oxygen and glucose (and other nutrients) but potentially also to important signaling molecules.

**Figure 1:**
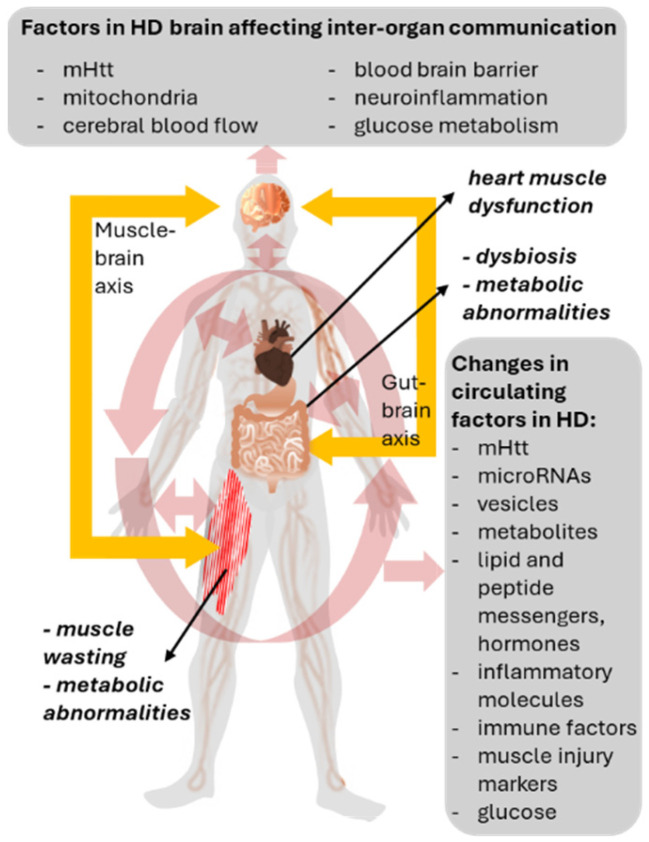
Overview of abnormalities in Huntington’s disease (HD) that likely lead to impaired inter-organ communication. A few peripheral abnormalities are indicated (in italics), for HD-related peripheral deficits in other organs, see [4]. The muscle–brain axis and gut–brain axis are highlighted because they are most relevant for the lifestyle interventions discussed below. mHtt, mutated Huntingtin.

With a lot of identified alterations in potential mediators of inter-organ communication in HD (summarized in Figure 1), the question arises, how those messengers can be targeted to hopefully modify or slow down disease progression, or attenuate HD-related symptoms. While many pharmacological and genetic strategies could be conceived for this purpose, and several currently explored approaches already target circulating substances (in particular mHtt [11,145]), here, we will focus on the potential of healthy lifestyles, because of their particular potential to improve inter-organ communication. Specifically, the opportunities and risks of dietary and exercise interventions for this purpose will be discussed in the next Section.

## 3. Healthy Lifestyles with Beneficial Metabolic Effects in HD

Although the number of CAG repeats is inversely correlated with the age of disease onset [146] and positively correlated with motor and non-motor symptoms [147], there is much individual variability in HD onset, severity of symptoms, and disease progression [148] and the duration of the manifest disease has been reported to be relatively independent of the number of CAG repeats in the *Htt* gene [149]. Genome-wide association studies have identified genes associated with DNA maintenance and repair, mitochondrial functions, oxidative stress, and proteostasis as major determinants [8,150] of the variability, which is further thought to be influenced by environmental and lifestyle factors, suggesting that modulation of these parameters can be protective for people with HD [151]. Lifestyle factors like exercise and a healthy diet may have overlapping or compensatory effects on cellular processes related to the mentioned genetic polymorphisms, since they may also affect DNA maintenance, mitochondria, oxidative stress, and proteostasis [152,153]. Specific metabolic vulnerabilities have to be considered for the application of lifestyle interventions in people with HD.

### 3.1. Dietary Approaches

A higher BMI can be associated with a more favorable disease progression [154] and smaller functional, motor, and cognitive impairments [36,41] in HD. Both symptomatic and asymptomatic HD patients were found to have significantly higher energy intake compared to controls (median: 5511 kcal/day and 3751 kcal/day vs. 2488 kcal/day, respectively) [39]. This increased caloric intake in carriers of HD-related CAG-expansion mutations already before symptom onset [38,39] in combination with the positive impact of higher BMI suggests that avoiding reductions in caloric intake is important. However, dietary interventions to increase BMI still likely do not benefit underlying disease processes that drive HD progression, since genetic markers of BMI do not determine disease onset: other factors probably drive disease progression and weight loss in parallel [155]. Which dietary interventions really are efficient in HD, is an open question. But several strategies will be briefly described here and their potential role in improving or re-establishing brain–periphery interactions will be discussed.

#### 3.1.1. Time and Calorie-Restricted Eating

In contrast to calorie-restricted eating, time-restricted eating refers to strategies in which food is taken up only during defined periods. Surprisingly, a dietary restriction regime (alternative day fasting, a form of intermittent fasting) not only improved glucose metabolism and delayed disease onset and death but also mitigated weight loss in the N171-82Q mouse HD model [156]. While similar benefits of alternative day fasting were shown in the YAC128 mouse HD model [157] and of food restriction (gradually decreasing food provision over several months) in the BACHD mouse model (expressing exon 1 of human mHtt with 97 mixed CAA-CAG repeats) [158], these mice exhibit higher body weight as compared to non-transgenic controls (not reflecting well the human disease), which was normalized by dietary restriction. R6/2 mice exhibit progressively decreasing body weight—similar to many HD patients—but even in this mouse HD model, some benefits of intermittent fasting have been described. Skillings and colleagues observed better temperature regulation and possibly attenuated deterioration of normal behavior at older age in R6/2 mice (mean CAG repeats of 250 ± 17 SEM) when food access was restricted only to the dark periods [159]. However, these authors also report reduced survival when this protocol was combined with environmental enrichment [159].

Intermittent fasting is thought to induce a coordinated cellular stress response, leading to cellular adaptation that increases cellular antioxidant and anti-inflammatory capacities, DNA repair mitochondrial functions, and mitochondrial and protein quality control [160]. Taken together, dietary restriction in mouse models of HD appears to mitigate some aspects of the disease, possibly by acting as a mild metabolic stressor that has the potential to enhance mitochondrial resilience and recalibrate metabolic homeostasis based on beneficial metabolic adaptations [152]. Additional stressors or stimulants (e.g., caused by environmental enrichment, or due to injury or severe metabolic stress in later stages of the disease) may counteract these adaptive capacities. Due to the reduction in blood glucose levels and compensatory upregulation of ketogenesis, prolonged fasting periods in time-restricted feeding contribute to a shift of metabolic fuels [161]. Beneficial effects on glucose and insulin homeostasis, intestinal health, and (primarily liver-derived) ketone-body signaling may contribute to better systemic metabolic health and an improved brain–periphery interaction.

Aside from the regulation of BMI and caloric or temporal eating behaviors, changing the diet composition has also been investigated as a strategy to improve metabolic health in HD, possibly again resulting in improved brain–periphery signaling. Eating behaviors are likely changed beyond the energy uptake in most HD patients. A recent study [39] on Cypriot people with symptomatic or asymptomatic HD patients, for example, found significantly reduced non-starch polysaccharide and fiber intake and various specific differences in asymptomatic HD-mutation carriers (including increased polyunsaturated fatty acid, cholesterol, and sodium intake) as compared to gender- and age-matched controls of the dietary reference intake [162]—which, however, did not necessarily reflect local and cultural dietary habits and therefore is difficult to interpret. Potentially changed dietary habits in HD leading to risks of dysbalanced nutrient intake could be associated with mediators of inter-organ communication. Several selected nutrients and metabolic pathways that may be targeted to modulate such circulating factors as presented in the previous chapter are discussed below.

#### 3.1.2. Kynurenine Pathway Metabolism and Inflammation

**Figure 2:**
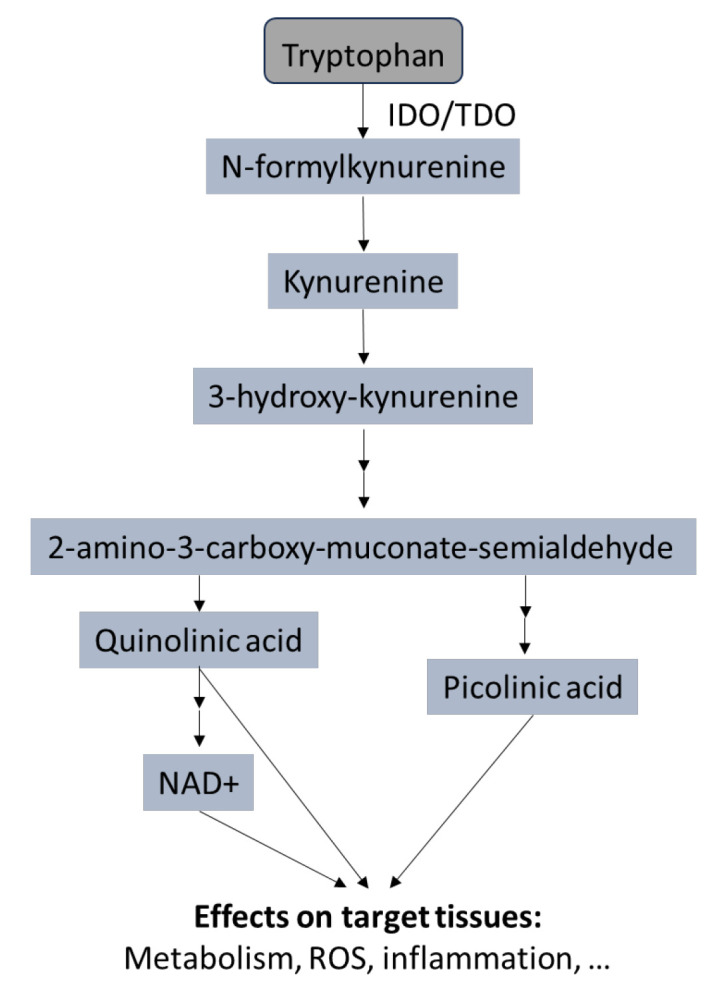
Tryptophan-kynurenine metabolism. Tryptophan is broken down by indoleamine-2,3-dioxygenase (IDO) or tryptophan 2,3-dioxygenase (TDO), yielding N-formylkynurenine that can be catabolized to kynurenine, which is further metabolized. Many of the resulting metabolites exert important effects, including on levels of reactive oxygen species (ROS) or inflammatory parameters, in different tissues, including the brain. NAD+: oxidized form of nicotinamide adenine dinucleotide.

Our literature search identified inflammatory and immune parameters that may be involved in perturbing brain–periphery communication. In a variety of chronic diseases and conditions that are related to immune activation and inflammation, significant alterations of the kynurenine pathway (KP) are apparent [163] and this may be the case also in HD [164]. The activated immune system can be detected by increased kynurenine (KYN) to tryptophan (TRP) concentrations. More than 95% of free TRP is degraded through the KP, and KYN metabolites, such as kynurenic acid and quinolinic acid, generated by this strategy, which can affect several body compartments, inducing local and systemic adaptations [165] (see Figure 2). Increased TRP breakdown rates have been noticed in the CSF of patients with neurodegenerative diseases, including HD [166], and a recent meta-analysis found that blood levels of TRP and KYN were significantly lower in HD patients compared to controls [164]. These potential changes in TRP and KYN pathways could be targeted in HD, but several studies also found no differences in HD [164], including one recent study comparing KYN metabolites in cerebrospinal fluid and plasma in people with HD, Parkinson’s disease, and controls [167]. Still, the potential effects of TRP and KYN on the regulation of the gut–brain axis and on inflammation deserve further consideration.

Accumulating evidence suggests that gut microbiota regulate KP metabolism and interference with the microbiome, thus, likely influencing the gut–brain axis [168]. TRP availability in KP metabolism can be modulated by changes in diet and lifestyle [169,170]. However, a diet rich in TRP is unlikely to be related to greater TRP availability to the brain as most individuals (even older individuals and patients) consume adequate amounts of this essential amino acid [171,172]. Moreover, TRP competes with other large neutral amino acids (LNAA) for transport across the blood–brain barrier, which limits TRP availability for the cerebral KP [173]. Therefore, the effect of diet on the TRP level in the brain depends on the TRP/LNAA ratio, which can be improved by a carbohydrate-rich diet by promoting insulin secretion and the absorption of LNAA by muscles. On the other hand, certain nutrients and bioactive compounds (such as antioxidants, omega-3 fatty acids, and probiotics) may suppress indoleamine-2,3-dioxygenase (IDO) activity, which degrades TRP into KYN, and slow down Th1-type immune activation cascades [174].

Dietary fat has a different effect on related enzymes of TRP metabolism. While a high-fat diet rich in saturated fatty acids was shown to increase IDO activity and impaired serotonin function in mice [175], a diet rich in n-3 long-chain polyunsaturated fatty acids (PUFAs) can increase TRP availability in the brain. A metabolomic analysis in 12 younger and 12 older adults observed significant reductions in circulating KYN levels following n-3 PUFA supplementation (3.9 g/day over four months) in older adults who exhibited modest elevations in KYN compared to younger [176]. An in vitro study found that n-3 PUFAs may be involved in regulating IL-1β, a pro-inflammatory cytokine that decreased neurogenesis in human hippocampal progenitor cells, by modulation of the KP, as n-3 fatty acids eicosapentaenoic acid (EPA, 10 µM) or docosahexaenoic acid (DHA, 10 µM) reduced levels of the neurotoxic quinolinic acid thereby prevented the IL-1β-induced reduction in hippocampal neurogenesis [177]. Furthermore, sufficient levels of EPA (≥2 g/day) increase the release of serotonin, and sufficient levels of DHA (1 g/day) influence serotonin receptor action [178]. Thus, there could be a direct effect of n-3 PUFAs on the disease [179]. Although the effects of n-3 PUFAs in HD are not yet well understood, omega-3 fatty acids exhibit anti-inflammatory, antioxidant, and neuroprotective properties that make them suitable candidates for the treatment of neurodegenerative disorders in general [180,181]. A clinical study revealed that daily supplementation of n-3 PUFAs (810 mg EPA and 4140 mg DHA) plus n-6 PUFAs (1800 mg gamma-linolenic acid and 3150 mg Linoleic acid) with antioxidant vitamins (0.6 mg vitamin A and 22 mg vitamin E), including gamma-tocopherol (760 mg), for 2.5 years significantly delayed disease progression in patients with early Parkinson’s disease [182]. Recent findings in older adults support a biologically plausible rationale whereby these nutrients work synergistically, and in a dose-dependent manner, to improve working memory, thereby reducing cognitive decline and dementia risk in later life [183].

Taken together, while it remains unclear whether circulating KYN metabolites play a crucial role in inter-organ communication in HD, targeting inflammatory parameters by targeting KYN-metabolism or with other dietary approaches (e.g., n-3 PUFAs) are interesting avenues to control dysregulated brain–periphery communication in HD.

#### 3.1.3. Oxidative Stress and Antioxidants

Antioxidant properties of vitamins and polyphenols (flavonoids) may also contribute by affecting the activities of enzymes involved in TRP metabolism. In vitro studies revealed that several phytocompounds can interfere with inflammatory signaling cascades including TRP breakdown [184]. Consequently, TRP availability in the brain rises. However, because these results were solely based on in vitro experiments, the effects of antioxidants on systemic TRP metabolism remain speculative. In addition, they most likely depend on the individual’s immunological state.

Oxidative stress is a crucial factor in HD [185] and circulating messenger molecules, such as exerkines, contribute to the regulation of oxidative stress [186]. Reactive oxygen species (ROS) are regulators of immune responses and inflammation in the brain, which together with imbalanced antioxidant defenses and overproduction of ROS (resulting in oxidative stress) are involved in neurodegeneration. A diet rich in antioxidants (polyphenolic compounds, carotenoids, vitamins E and C) can decrease cellular oxidative stress and exert neuroprotection [187]. Accordingly, vitamin E supplementation significantly improved memory, cognition, learning, motor function, and brain markers associated with neuroregeneration and neuroprotection in experimental models of neurodegenerative diseases [188]. Both vitamin C and vitamin D have been shown to ameliorate motor abnormalities models of HD in HD patients, as recently reviewed [189], and a high-dose vitamin E treatment (3000 IU of d-α-tocopherol/day for 1 year) was moderately effective in slowing down the progression of symptoms in early HD stages [190]. In addition, several flavonoids can protect striatal neurons, thereby ameliorating symptoms in HD models [191]. Moreover, polyphenol-rich dietary patterns counteract gut microbiota dysbiosis [192] and intestinal permeability [193], which is strictly associated with chronic activation of the immune system. Polyphenol compounds may exert neuroprotective actions after biotransformation by specific gut microbiome metabotypes and intestinal mucosa absorption [194], recently reviewed by Ticinesi et al. [195].

#### 3.1.4. Probiotics and Other Modulators of the Gut–Brain Axis

The intestinal microbiome modulates the risk of many age-related chronic syndromes, including neurodegenerative diseases [196]. Nutritional strategies to counteract metabolic challenges are especially relevant for people with HD who suffer from gastrointestinal dysfunction [98]. Preclinical findings in mice (R6/1) suggest that a high fiber (10%) containing diet reduces pro-inflammatory bacteria in gut microbiota and improves gastrointestinal function in HD [197]. It also improved cognition and affective behaviors in HD model mice [197], suggesting a diet high in fiber can correct impaired gut–brain crosstalk in HD. In addition, probiotics can modify the population of gut microflora and have been shown to increase some aspects of mucosal and systemic immunity in healthy humans [198]. Yet, in a recent randomized clinical trial of a 6-week probiotic intervention in 41 patients with HD, probiotics did not ameliorate gut dysbiosis or clinical features of HD [199]. Importantly, the modulation of the gut microbiome is a complex process and depends on the resulting microbial composition, which may have to be further optimized for HD to achieve promising results as reported for other neurodegenerative diseases [200]. The specific metabolic abnormalities in HD may require a corresponding microbiome composition to re-establish a functional gut–brain axis.

#### 3.1.5. Mediterranean Diet

The Mediterranean Diet consists of a high consumption of plant-based foods, legumes, nuts, whole grains, fish, and olive oil, which is one of the richest sources of monounsaturated fatty acids (MUFAs), and provides nutrients, phenolic compounds, and antioxidants that benefit brain health [201,202]. The NU-AGE study recently demonstrated that a 1-year Mediterranean Diet intervention was able to modulate specific components of the gut microbiota with an increase in short-chain fatty acid (SCFA) production that was associated with a reduction in risk of frailty, improved cognitive function, and reduced inflammatory status [203]. SCFAs, and particularly butyrate, are generated through the fermentation of dietary fibers and other components (such as nitric oxide, ammonia, and ethanol) by gut bacteria and exert a wide range of metabolic functions [204], but also influence neural activity and brain function [205,206]. Consuming a Mediterranean Diet rich in fibers, MUFAs, and PUFAs with a high polyphenol content is associated with an increase in beneficial microbial species, such as *Lactobacillus*, *Bifidobacterium*, *F. prausnitzii*, *Lachnospira*, and *Prevotella*, while reducing levels of *Firmicutes*, *Ruminococcus*, and *Escherichia coli* [207].

Despite the wealth of evidence demonstrating a protective role of the Mediterranean Diet in neurodegeneration, only a few studies have investigated the effect of the Mediterranean Diet on HD. Overall, the evidence suggests an improvement in the cognitive and motor scores and a better quality of life in people with HD who report high Mediterranean Diet adherence [208]. Moreover, a higher consumption of milk and dairy products and caffeine consumption greater than 190 mg/day seem to be associated with an earlier age of onset. Regarding milk/dairy product consumption this association is based on self-reported data from an early study, the authors of which doubt any biological relevance [209]. The European Huntington Disease Network (EHDN) Registry study demonstrated that moderate-to-high Mediterranean Diet adherence, characterized by a higher intake of MUFAs/saturated fatty acids, was associated with improvements in total functional capacity and cognitive scores compared to low Mediterranean Diet adherence [210]. Possible mechanisms linking the Mediterranean Diet to gut microbiome and HD progression may involve the capacity of the Mediterranean Diet to stimulate the synthesis of SCFAs and to reduce inflammation and gut permeability, as well as the capacity to transform dietary (poly)phenols into bioactive compounds, such as urolithin, genistein, or resveratrol, released after intestinal biotransformation [211].

Overall, dietary strategies can improve inter-tissue communication in HD by regulating the gut–brain axis, redox-homeostasis, and inflammation. They can also be applied to modulate glucose levels and reduce unintentional weight loss. Importantly, the causalities between dietary choices, body composition, gut microbiome composition, BMI, appetite, and HD-related motor and non-motor symptoms are poorly understood. Strict dietary regimes can be risky for people with HD, and body weight changes and symptoms have to be closely monitored. Thus, while generally promising, more research on the outcomes of specific dietary interventions and/or strategies to modulate the gut–brain axis in HD, and personalized, stringently monitored approaches are required. A better understanding of particularly the dysregulation of insulin and glucose metabolism and of appetite-related hormones (such as ghrelin and leptin)—as well as of the translational validity of these factors from animal studies—in HD are crucial for the design of optimized diets for people with HD.

### 3.2. Exercise

Exercise exerts well-established benefits on the brain [212] and the muscle–brain axis and myokines appear to be strongly involved for example in the regulation of cognition, as recently summarized for pre-clinical findings [3]. Accordingly, some studies also suggest improvements in cognitive function in HD. A recent study found amelioration of motor learning even after one aerobic exercise bout (20 min moderate intensity cycling) in presymptomatic and early-manifest HD [213]. On the other hand, a recent systematic review did not find pronounced effects of physical exercise on cognition in HD, while cognitive exercise was more efficient [214].

The therapeutic value of exercise in HD has been investigated in both experimental model systems and human subjects [215]. In the latter, exercise programs are often embedded within multi-modal therapies that might include occupational therapy, speech therapy, cognitive training, and respiratory exercises on-site under trained supervision, and/or home-based exercise performed independently but with general guidance provided [215]. Systematic reviews and meta-analyses aiming to evaluate the efficacy of exercise in its different forms and under these varied settings largely support benefits for HD but recommend larger controlled trials to further establish efficacy [214,216,217,218]. Given the multi-modal structures of therapy in many trials, it remains challenging to attribute any durable clinical response to exercise itself as a stand-alone therapy, or to the other therapies being performed concurrently. Therefore, in this chapter, we will summarize some published exercise interventions with promising findings. However, before that, it is important to discuss the caveats of exercise in HD, considering metabolic and peripheral aspects of the disease.

#### 3.2.1. Risks and Safety of Exercise Interventions for HD

While exercise, alone or as part of multi-modal therapy, has been reported to offer some clinically meaningful benefits on motor functions as well as on the overall quality of life in smaller cohorts of HD [214,216,217,218], exercise interventions in HD need to be carefully designed, considering individual capacities and limitations [219,220].

In the N171-82Q mouse model of HD, voluntary wheel running was used to examine whether aerobic exercise activity could mitigate HD features [221]. Wheel running began before the appearance of overt HD symptoms, enabling the evaluation of whether aerobic exercise could delay the onset of HD or reduce the disease burden [221]. Interestingly, aerobic exercise appeared to accelerate the onset of HD [221]. This was evidenced first by HD symptoms appearing significantly earlier in runners compared to sedentary controls [221]. Runners also had the most pronounced locomotor deficits and, significantly, lower striatal volume compared with sedentary HD mice, but this did not affect lifespan [221]. The authors suggested the observed HD deficits may relate to unknown factors released into the circulation due to a metabolically compromised HD system unable to cope with the energetic demands of exercise [221]. In practical terms, these observations imply the need for appropriate management of exercise in HD, and to comprehend those conditions in HD individuals where exercise is contraindicated.

Corrochano et al. similarly suggested that exercise may be contraindicated in HD under some conditions [222]. Using different mouse models of HD and HD-related pathology, they proposed that interventions such as aerobic exercise, which impose high energy demands, may be detrimental in HD due to significant energetic stress already present in HD skeletal muscle [222]. Imposing additional metabolic stress in already compromised mouse muscle then contributes to systemic abnormalities that worsen HD disease burden [222]. This framework was based on two sets of experiments. In the first, an unbiased genetic screen was performed in an HD mouse model (based on N171–82Q mice) to identify disease modifiers of HD [222]. The screen identified a mutation in Scn4a, a voltage-gated sodium channel enriched in skeletal muscle [222]. This mutation enhanced the HD phenotype including earlier onset of tremors and reduced survival [222]. Intermittent paralysis was a new trait attributed to the Scn4a mutation independently of the HD transgene [222]. Further, these HD mice carrying the Scn4a mutation had lower body weight, increased whole-body energy expenditure, and loss of lean and fat mass consistent with cachexia [222]. Phenotyping of skeletal muscles showed these mice had a metabolic profile similar to that induced by aerobic exercise training, despite the worsening of the disease [222], suggesting a potential link between exercise-induced energy stress in muscle and HD pathology. To examine this further, when HD mice were subjected to forced training using the rotarod 30 min/day for 5 days/week plus voluntary running in the home cage, HD mice had greater lean mass loss and shorter survival compared with sedentary HD counterparts [222]. Overall, the authors concluded that energetically demanding physical activity could be detrimental in HD due to metabolic deficits in peripheral tissues such as skeletal muscle [222]. More broadly, it suggests that careful consideration may need to be given to the heterogeneous nature of HD in relation to exercise, particularly the metabolic state of peripheral tissues such as skeletal muscle before undertaking exercise. Regarding the modulation of inter-organ crosstalk, these findings may indicate the continuous release of molecular messengers from HD muscle, interfering with the signaling efficiency achieved by the regulated peaks of such a release during exercise in healthy muscle. This would be consistent with increased circulating muscle injury markers in the blood of different HD mouse models (see Table 2, [73]). Circulating markers for neuronal damage, such as neurofilament light protein, are also higher in people with HD (for review see [11]) and interestingly, serum neurofilament light protein could be reduced by exercise in other diseases, such as in multiple sclerosis of the relapsing-remitting type [223]. This suggests that exercise can re-establish healthy levels of circulating injury markers, from the brain and maybe from skeletal muscle.

In humans with HD, the discussed findings of increased lactate levels during exercise and reduced anaerobic threshold (see Table 2, [75]) also indicate reduced exercise tolerance in people with HD. In line with these findings, a case report documented evidence of metabolic myopathy on muscle biopsy in a high-level marathon runner, who was a presymptomatic HD mutation carrier [224]. With time, exercise intolerance developed and elevated creatine kinase (consistent with muscle damage and myopathy) and mitochondrial abnormalities were detected; all preceding principal HD symptoms [224]. This case study offered unique insight because it suggested associations between a candidate disease modifier in habitual vigorous exercise, peripheral traits (skeletal muscle abnormalities), and subsequent central alterations manifesting as chorea in at-risk HD gene carriers. One interpretation might be that vigorous exercise is not favorable in HD, as suggested by some [225], such that strenuous endurance exercise for an extended time led to HD-associated metabolic myopathy in this at-risk individual. Another view is that muscle metabolic dysfunction occurs early, during presymptomatic HD [224]. If muscle metabolic impairment occurs before symptomatic HD and reflects exercise intolerance, screening for signs of muscle metabolic impairment may aid decision making in HD regarding risks versus benefits of vigorous exercise. Lastly, the strenuous, high exercise volume performed by this at-risk HD individual would probably not be used in a therapeutic exercise program. Exercise programs that tailor volume and intensity to the individual, with regular surveillance and management of workload, could provide an avenue by which to best maximize benefits and manage risks of therapeutic exercise in heterogeneous HD.

One common aspect of possible significance in the above-mentioned mouse experiments and the case study is biological sex. In the N171-82Q transgenic mouse model of HD, adverse outcomes associated with wheel running were reported for males [221]. In the study by Corrochano et al. [222], focus was placed on male mice in most experiments because the disease phenotype was most prominent in males. The case report of exercise intolerance, muscle pain, and metabolic myopathy before detection of chorea also occurred in a high-risk male [224]. These findings in males suggest that sex may interact with a compromised neuromuscular system and other biological variables to influence responsiveness to exercise in HD.

#### 3.2.2. Standalone Exercise with Clinical Benefits

Discussed below are selected studies that investigated standalone exercise therapy in HD and thus provide the most insight into the isolated impact of exercise. A pair of studies by the same group examined the impact of 6 months of aerobic training in male people with HD and healthy male controls [226,227], while another examined combined aerobic and resistance exercise training [228]. Appropriate recovery periods were interspersed between thoughtfully designed, progressive training programs, which were consistent with recommended practices and principles of exercise adaptation [229,230].

In the first study of aerobic training, outcomes included cognitive and motor function and cardiovascular performance [226]. The intervention was progressive with respect to workload and consisted of the following design: 3 blocks that lasted 10, 8, and 6 weeks each. In the first block, participants performed cycling exercise 3× per week at 65% of VO2peak for 30 min. This block was designed for moderate-intensity exercise of extended duration to build a strong aerobic base that could accommodate greater strain in later blocks. In the second block, high-intensity interval training (HIIT) was performed 3× per week; the HIIT comprised 4 × 4 min of cycling at a power output corresponding to ~95% maximum heart rate. This type of exercise elicits high cardiovascular and muscular strain in a short period and can produce adaptations typically associated with longer-term endurance training [231,232]. In the last block, 2 HIIT sessions and 1 fixed-intensity cycling session were performed. Each block was separated by 1 week of recovery, where the workload was reduced to permit restoration while also maintaining an elevated level of activity (i.e., active recovery). Training stabilized motor function in HD patients, as evidenced by deficits measured in the UHDRS at the start of training but did not improve baseline values [226]. HD patients also increased their maximal aerobic capacity to a similar degree as healthy controls, and no adverse events were reported [226]. Together these findings suggest HD status is not a barrier to achieving positive adaptations with appropriately designed endurance-type exercise. In addition, the training program might have slowed down disease progression, although no clinical improvements compared to baseline were achieved.

In the second study of aerobic training by this group, muscle biopsies were collected and analyzed to evaluate mechanisms of skeletal muscle adaptation to aerobic training in HD [227]. The same endurance training program as described above [226] was implemented in male HD patients. Assessed were mitochondrial enzyme activity, and in situ respiratory capacities and fiber capillarity. Endurance training significantly increased the activities of citrate synthase, complex II, and complex III to a similar extent in both HD and controls [227]. Of note, the activity of complex I, a principal site of electron input into the respiratory chain, was decreased at the start of training versus 6 weeks prior [227], reflecting an HD-associated impairment of muscle mitochondrial function. No further deficits occurred, however, as a result of the training [227], suggesting that aerobic training slows progressive HD-related declines in oxidative metabolism. In agreement with improved mitochondrial enzyme activity, training enhanced mass-specific oxidative phosphorylation capacities in fiber bundles of HD patients [227]. Training also improved fiber capillarity to a similar degree in HD and controls [227]. Collectively, these findings indicate that mechanisms of muscle energy metabolism respond favorably to endurance training in HD [227]. Similar degrees of improvement in HD and control muscle [227] might also suggest no major intrinsic defects in HD muscle that would lead to exercise resistance in HD. An important factor to weigh these findings against is the lower disease severity of the cohort, with mean UHDRS motor scores of 18 and a mean total functional capacity score of 11 [227]. Disease severity at the onset of exercise training, therefore, may be an important determinant of favorable response, with lower disease severity potentially more amenable to training-induced benefits.

In contrast to aerobic exercise activities described above, the high mechanical overload characteristic of resistance exercise is relatively less studied as stand-alone therapy [215]. Resistance exercises appear most often within multi-disciplinary programs that also include aerobic exercise and non-exercise therapy [215,233,234,235]. In one randomized phase II trial that excluded non-exercise therapy, 12 weeks of combined aerobic and resistance training was examined [228]. Of the 31 participants (50.4 ± 11.4 years, 15 females), 16 (UHDRS total motor score 32.4 ± 15.5) were randomly assigned to the exercise condition. One of the 3 weekly sessions took place under the supervision of an exercise physiologist or physical therapist while the remaining were home-based and performed independently. In the supervised session, aerobic exercise was performed first and consisted of cycling at 55–75% of the age-predicted maximum heart rate, an intensity corresponding to moderate–high, for a duration beginning with 20 min and progressing up to 30 min [228]. After cycling, resistance exercises were performed in a circuit fashion that targeted knee extensors, knee flexors, ankle extensors, and latissimus dorsi. Initial loads corresponded to 10 repetitions maximum, with progression to 2 sets of 8–12 repetitions at 60–70% of the one-repetition maximum (i.e., a moderate/moderate–high resistance load) [228]. Moderate effect sizes for the intervention on cognitive function and walking performance were reported [228], suggesting the benefits of combined aerobic and resistance exercise training in this cohort of HD. Because the design included both types of exercise, it is difficult to attribute outcomes to a specific training modality. How different combinations of exercise programming variables affect clinical outcomes requires systematic, controlled trials to guide exercise program design for HD groups.

In another report, the impact of a 6-month aerobic training intervention versus an active stretching control on MRI-determined brain structure was studied in pre-manifest HD [236]. Participants in the aerobic training group performed 3 sessions per week of moderate–vigorous walking at 70% of maximum heart rate [236]. Progression was implemented by gradually extending the length of sessions from 15 to 50 min per session during weeks 1–6, reflecting a goal of 150 total minutes per week in accordance with American College of Sports Medicine guidelines [236]. Improvement in aerobic capacity was more pronounced in the aerobic training group compared with active control [236]. While no clinical differences were found, greater increases in aerobic capacity were associated with reduced atrophy of the hippocampus, thalamus, pallidum, and cerebellar cortex [236]. This suggests that exercise programs that efficiently improve aerobic capacity are particularly neuroprotective. Exercise programming strategies that safely assign workloads sufficient to maximize aerobic capacity, therefore, might be most beneficial for maintaining the integrity of brain regions involved in HD pathology.

**Figure 3:**
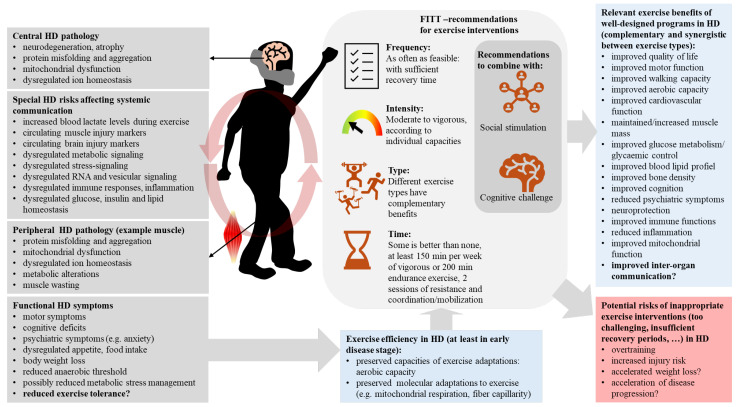
Factors impacting the safety and benefits of exercise in Huntington’s disease (HD). See text for details and references. The FITT (frequency, intensity, type, time) recommendations are based on [239] and the WHO guidelines on physical activity and sedentary behavior [240].

Overall, complex relationships between metabolic impairments and therapeutic exercise impact safety and responsiveness to exercise in HD. Configuration of the exercise stimulus such as type of exercise, intensity, and duration determines outcomes. These nuances need careful consideration and additional research for the development of appropriate programmed exercises to provide durable clinical responses while managing risk. Still, the multifaceted benefits of regular exercise are huge (Figure 3), and the creation of customized exercise interventions can produce immense general benefits [237,238] and very relevant advantages in HD.

## 4. Conclusions

It is likely that brain pathology and peripheral symptoms influence each other in HD and modulate disease progression. Therefore, strategies that ameliorate either central or peripheral deficits are thought to benefit HD [30]. In particular, the re-establishment of healthy (metabolic) communication between the brain and other organs may be promising. This is difficult to achieve by pharmacological interventions but both exercise and dietary strategies can improve inter-tissue communication, as discussed in the present review.

However, specific abnormalities in people with HD demand caution in the prescription of diet and exercise. The modulation of gut–brain interaction, e.g., by targeting the composition and function of the microbiome is a promising, emerging research field—however, the low number of available longitudinal studies and limited knowledge on the translational validity of animal studies presently are major barriers [93].

Exercise is a potent strategy to prevent many chronic diseases and to improve inter-organ signaling, in particular of the muscle–brain axis [239]. Appropriate, personalized programs likely are also highly beneficial in HD; however, disease-related limitations in exercise tolerance and metabolic deficits need to be considered.

It will be an important future endeavor to investigate the translational validity of animal experimentation for people with HD. Specifically, intermittent fasting approaches that showed promise in murine HD models may not be appropriate in humans with HD, especially if the HD models—unlike humans with HD—are associated with increased body weight [156,157]. Also, the detrimental outcomes of exercise in HD mouse models do not seem to correspond with the results of (customized) exercise programs in people with HD [221,222].

Taken together, dysregulated brain–periphery communication is likely a cause or consequence of HD-related disease processes that lead to pathology, also outside of the central nervous system. Re-establishment of efficient inter-organ communication therefore may either modify disease progression or improve symptoms. The combinations of customized dietary interventions and/or exercise programs appear to be capable of improving such whole-organism communication in HD; however, such strategies require careful design and monitoring.

## Figures and Tables

**Table 1:** Factors related to mHtt, inflammation, and the immune system.

Species	Subjects/Model	Main Findings	References
mHtt			
Mouse	Female zQ175 HD model mice in parabiosis with wild-type mice	mHTT was found in the plasma and circulating blood cells of wild-type mice and mHTT aggregates in organs like liver, kidney, muscle, and brain (including vascular abnormalities), suggesting that mHTT is transported in the blood and can induce pathology in remote organs.	[53]
Mouse	Female zQ175 HD model mice in parabiosis with wild-type mice	mHTT aggregation and a compromised BBB were observed in wild-type mice sharing their circulating system with HD mice. Ablation of the hematopoietic niche did not significantly affect these results.	[54]
Inflammatory factors, immune system			
Human	Different cohorts of HD patients and controls used for the individual experiments (for detailed information see Supplementary Materials in [55])	HD myeloid cells produced increased inflammatory cytokines due to mHtt and its effects on the NFκB pathway. Reducing Htt with small interfering RNA particles in HD monocytes/macrophages, reversed the excessive cytokine production and associated transcriptional changes.	[55]
Human	12 people with manifest HD (42.4 ± 1.7 years, 4 female, disease duration 2–13 years, 11 controls (47.0 ± 12.0, 4 female)	Immunoglobulin A, soluble tumor necrosis factor receptor, interleukin-2-receptor, neopterin, and complement component C3 increased in HD serum and tryptophan decreased. These changes were correlated to cognitive deficits.	[56]
Fly	*Drosophila* expressing human Htt-Q93 exon 1 in neurons	Neuronal mHtt-Q93 caused elevated ROS levels in circulating immune cells, reduced immune cell numbers, and perturbed immune function.	[57]
Fly	*Drosophila* expressing human Htt-Q93 in hemocytes (insect immune cells)	mHtt expression in hemocytes did not impair motor function but compromised the immune system, leading to greater infection susceptibility.	[58]
Mouse	Female YAC128 HD model mice	Unlike in skeletal muscle and brain, transcriptional drifts in splenic T-cells suggest accelerated aging in YAC128 mice.	[59]
Mouse	3-nitropropionic acid model of HD in 12-week-old C57BL/6 mice	Alkylated resveratrol prodrug improved inflammation (reduced serum interleukin-6 levels) and delayed motor symptom onset and weight loss.	[60]
Mouse	R6/2, HdhQ150 knock-in and YAC128 mouse models of HD	Myeloid cells from the spleen and blood cells from HD model mice produced increased inflammatory cytokines but not bone marrow CD11b(+) cells. Greater phagocytosis was observed in R6/2 macrophages, reflecting observations in HD patients [55].	[61]

Notes. See main text (Section 1.2) for a description of animal models. BBB, blood–brain barrier, CD11b, cluster of differentiation molecule 11B; HD, Huntington’s disease; HdhQ150, knock-in mouse model of HD expressing mutated Huntingtin with multiple glutamines (Q); Htt-Q93, drosophila model of HD with mutated Huntingtin expressing multiple glutamines (Q); mHtt, mutated Huntingtin protein; NFκB, nuclear factor ‘kappa-light-chain-enhancer’ of activated B-cells; ROS, reactive oxygen species; YAC128, yeast artificial chromosome 128 (mouse model of HD expressing human full-length mutated Huntingtin); zQ175, murine knock-in HD model with a chimeric murine/human expanded glutamine (Q) repeat sequence in the mouse endogenous Huntingtin protein.

**Table 2:** Circulating metabolites and muscle injury markers.

Species	Subjects/Model	Main Findings	References
Muscle injury markers			
Mouse	R6/2 and Q175 HD model mice	Increased levels of muscle injury markers in HD mouse serum: skeletal Troponin I (sTnI), fatty acid binding protein 3 (FABP3), and Myosin light chain 3 (Myl3). In HD mice, genes related to muscle contractility were downregulated and components of the nuclear factor ‘kappa-light-chain-enhancer’ of activated B-cells (NfκB) were upregulated.	[73]
Circulating metabolites			
Human	7 people with HD (3 female, range 40–66 CAG) with fast progression, 13 (6 female, range 41–49 CAG) with absent progression (range 24 to 67 years for both groups)	Decreases in several plasma metabolites (related to oxidative stress, inflammation, nitric oxide/urea/glucose metabolism, polyamines, AMPK signaling) were associated with faster progression.	[74]
Human	15 people with symptomatic (6 female, 48.2 ± 10.2 years, 45.3 ± 3.2 CAG) and 10 with presymptomatic (3 female, 37.6 ± 6.7 years, 43.8 ± 2.5 CAG) HD, 25 controls (9 female, 43.7 ± 10.6 years)	Higher plasma levels of lactate in people with HD were correlated with a lower anaerobic threshold than controls.	[75]
Mouse	Male R6/1 mouse model of HD (4 to 12 weeks old)	Early gut microbiome perturbance and gut dysbiosis and elevated butanoate metabolism pathway in 12-week-old R6/1 mice, modest changes in plasma metabolome.	[76,77]

Notes. AMPK, adenosine monophosphate-activated protein kinase; CAG, Cytosine, Adenine, Guanine—here abbreviated for CAG repeats; EV, extracellular vesicles; HD, Huntington’s disease.

**Table 3:** Circulating lipid and peptide messengers, hormones.

Species	Subjects/Model	Main Findings	References
Human	42 manifest HD gene expansion carriers (60% females, UHDRS motor score 37.5 (30.5–71), and 30 healthy controls	Plasma levels of anandamide and 2-arachidonoylglycerol did not differ between groups and were not correlated to UHDRS or other clinical scores.	[102]
Human	15 people with pre-manifest (46.8 ± 2.1 years, 42.3 ± 0.1 CAG repeats), 8 with manifest HD (57.6 ± 4.1 years, 42.5 ± 0.1 CAG repeats), 16 familial, 10 pre-manifest, and 5 manifest age- and sex-matched controls.	Decreased levels of circulating growth factors (growth hormone, prolactin), total cholesterol, HDL-C, and LDL-C and perturbed levels of ghrelin, glucagon, and amylin in pre-manifest and manifest HD. Increased C-reactive protein in pre-manifest HD subjects.	[103]
Human	Progression groups (manifest HD): 21 low (35.6 ± 7.2 years, 41.4 ± 1.5 CAG), 47 medium (44.9 ± 10.1 years, 41.8 ± 2.2 CAG) and 52 high (49.2 ± 10.9 years, 42.8 ± 2.3 CAG), 30 controls (45.8 ± 11.4 years), men and women	Plasma 24S-hydroxycholesterol levels progressively decreased with an increasing disease progression score and correlated with several clinical markers, including striatal volume and UHDRS total motor score.	[104]
Human	96 people with manifest HD (stage 1–3), 33 HD gene-positive pre-manifest subjects (38–55 CAG repeats), 62 controls, men and women	Levels of the brain-generated cholesterol metabolite 24S-hydroxycholesterol, but not cholesterol itself, were reduced in people with symptomatic HD as compared to presymptomatic and control groups. Circulating 24S-hydroxycholesterol levels correlated with striatal volume decrease.	[105]
Human	Presymptomatic: 10 women (37 ± 2 years), 7 men (38 ± 4 years), Clinical stage I/II: 16 women (46 ± 3 years),12 men (43 ± 2 years), Clinical stage III: 19 women (47 ± 3 years) and 10 men (51 ± 3 years), Clinical stage IV: 3 women (53 ± 6 years), 5 men (50 ± 5 years), Controls 40 women (46 ± 3 years), 28 men (43 ± 3 years)	Increasing urinary cortisol with disease progression.	[44]
Human	15 HD mutation carriers (48.9 ± 3.2 years, 9 female, UHDRS 43.8 ± 5.5), 20 healthy controls undergoing orthopedic surgery (46.2 ± 4.1 years, 9 female)	Higher ghrelin and lower leptin levels in plasma of people with HD. No differences to controls in cerebrospinal fluid samples. No significant differences for insulin, glucose, insulin-like growth factor 1, or growth hormone. No correlations with disease duration were found.	[106]
Mouse	Female R6/2 HD model mice	During the symptomatic stage (12 weeks old), plasma ghrelin levels were reduced in R6/2 mice, and expression of several components of the ghrelin axis and circadian rhythms were perturbed. Chronic ghrelin treatment attenuated metabolic and drinking/resting behavior impairments.	[107]
Mouse	Heterozygous Q175 HD model mice	Reduced 24-hydroxy-cholesterol levels in plasma of Q175 mice reflect changes in cholesterol metabolism in the brain.	[108]
Mouse	Male and female R6/2 HD model mice	Increased circulating corticosterone levels in R6/2 mice (similar to increased cortisol levels in HD patients. Adrenalectomy and normalization of corticosterone levels improved metabolism (indirect calorimetry) and skeletal muscle wasting. In female R6/2 mice, it also attenuated brain atrophy and mHtt pathology.	[109,110]
Mouse	Male and female BACHD model mice	BACHD mice have increased serum leptin, insulin, and insulin-like growth factor 1, and developed impaired glucose metabolism and pronounced insulin and leptin resistance (these effects could be reproduced by targeted overexpression of mHtt in the hypothalamus but could not be abolished by inactivation of mHTT in leptin receptor-expressing neurons).	[111,112]
Mouse	Male and female R6/2 and CAG140 mice	Leptin and adiponectin levels were reduced in HD mouse plasma.	[113]
Mouse	R6/2 HD model mice	Levels of circulating glucose, insulin, ACTH, and corticosterone were higher and of corticotrophin-releasing hormone lower in HD mice; the adrenal cortex was enlarged. These events may explain muscular atrophy, reduced bone mineral density, abdominal fat accumulation, and insulin resistance in R6/2 mice. Increased cortisol levels were confirmed in HD patients in the same study (see above).	[44]

Notes. ACTH, adreno-corticotrophic hormone; BACHD, bacterial artificial chromosome-mediated transgenic HD, CAG, Cytosine, Adenine, Guanine—here abbreviated for CAG repeats; Corticosterone, rodent homolog of human cortisol; HD, Huntington’s disease; HDL-C, high-density lipoprotein-cholesterol; LDL-C, low-density lipoprotein-cholesterol; UHDRS, Unified Huntington Disease Rating Scale (from 0 to 124, higher scores indicate a higher level of motor function impairment. Early stage: <25, middle stage: 25–50, late stage: >50) [114], Q175, mouse HD model containing human mutated huntingtin with an expanded glutamine (Q) repeat within the native mouse huntingtin gene.

**Table 4:** Circulating RNAs and extracellular vesicles.

Methods	Subjects/Model	Main Findings	References
Studies in humans			
Proteomics and RNAseq on EVs isolated from human plasma	22 people with presymptomatic HD; 20 with early-manifest HD, 24 controls (more details in [128])	Although major EV characteristics (size, concentration) were similar between groups, differences in RNA content yielded good sensitivity to distinguish the groups. More EVs of small size were detected in the HD groups. The patterns of dysregulated RNAs suggest the liver to be the main organ releasing HD-specific EVs.	[129]
Transcriptomics and proteomics on skeletal muscle, skin (fibroblasts) and adipose tissue	21 people with presymptomatic HD (31.2–56.5 years, 10 females, 40–48 CAG repeats); 20 with early-manifest HD (35.4–58.9 years, 41–50 CAG repeats, 10 females), 20 controls (31.6–55.9 years, 10 females)	Robust transcriptomic and proteomic dysregulation depending on disease stage, with inflammation, energy metabolism, and EVs being confirmed as crucial factors in peripheral HD pathologies; e.g., peroxisome proliferator-activated receptor alpha dysregulated in presymptomatic HD muscle and early HD adipose tissue. TBC1D3D gene expression (involved in EV regulation) was downregulated in all analyzed tissues in presymptomatic HD and restored in early HD.	[128]
Exploratory microarray study of whole noncoding RNA expression profiles in plasma	9 people with HD (48.25 ± 10.47 years; 5 female), 8 healthy controls (49.17 ± 11.79; 6 females) 5 psychiatric patients (50.25 ± 11.47; 3 female), confirmation in 23 symptomatic HD, 15 patients with pre-manifest HD, and controls	Higher levels of SNORD13 in HD patients were correlated to disease duration and symptoms and affected factors relevant to HD pathogenesis. The authors suggest SNORD13 as a peripheral marker for cerebral HD pathology.	[130]
Exploratory microarray study of whole noncoding RNA expression profiles in plasma	9 people with HD (48 ± 10 years old; 5 female), 8 healthy controls (49 ± 12; 6 female) 5 psychiatric patients (50 ± 11; 2 female), confirmation in 33 HD gene carriers, and controls (healthy and psychiatric patients)	Downregulation of hsa-miR-98 (−1.5-fold) and upregulation of hsa-miR-323b-3p (+1.5-fold) in HD.	[131]
Human: 12 months of a customized HD diet, biochemical analysis of blood samples, cognitive and clinical testing	11 people with manifest HD (5 female, 49,0 ± 10,1 years old) and BMI ≤ 18 kg m^−2^ or unintentional weight loss	Unintentional weight loss was prevented by a customized diet in all participants: fat mass and blood leptin increased. Cognition (6/11) and motor function (3/11) improved in some participants only. Several circulating miRNAs (that were previously reported to be increased in HD) were downregulated due to the customized diet.	[132]
Mouse study			
Immunohisto-/cytochemistry, western blots, flow cytometry	R6/2 and zQ175 HD model mice in heterochronic parabiosis with wild-type HD mice (combinations of ages from 6 to 30 weeks)	Heterochronic parabiosis improved markers of mitochondrial biogenesis and cell death, reduced weight loss, and increased cognitive function and survival in HD.	[133]

Notes. BMI, body mass index, CAG, Cytosine, Adenine, Guanine—here abbreviated for CAG repeats; EV, extracellular vesicles; HD, Huntington’s disease; TBC1D3D, TBC1 Domain Family Member 3D; SNORD13, U13 small nucleolar RNA.

**Table 5:** Changes in cardiovascular and neurovascular parameters.

Species	Subjects/Model	Main Findings	References
Brain blood flow and neurovasculature			
Human	18 pre-manifest (44.3 ± 11.3 years, 41.9 ± 1.8 CAG repeats, 12 female) and 21 manifest HD gene carriers (49.9 ± 12.6 years, 44.6 ± 4.1 CAG repeats, 14 female) and 16 controls (48.12 ± 11.02 years, 10 female)	Bilaterally decreased cerebral blood flow in caudate and putamen correlated with worse motor and cognitive symptoms and with markers of neurodegeneration.	[28]
Human	15 pre-manifest and manifest HD patients (3 females, 27–77 years old, 40–44 CAG repeats) and 14 matched controls, post-mortem tissues from 22 HD patients (43–54 CAG repeats) and 9 controls	Despite relatively preserved larger cerebral blood vessel morphologies, arterial cerebral blood volumes were decreased in cortex in HD and blood vessel density increased in HD putamen. Markers for blood–brain barrier function indicate leakage in HD, which was confirmed by increased extravascular fibrin deposition. In vivo MRI data suggest blood–brain barrier deficits increase with disease progression. Similar results were obtained in R6/2 mice, published in the same paper.	[19]
Human	17 people with early-manifest HD (50.3 ± 5.5 years, 43.8 ± 1.7 CAG repeats), 41 controls	Cerebral blood flow strongly reduced in HD in specific cortical and subcortical areas. Cerebral blood flow was associated with cognitive performance (Stroop test).	[23]
Human	18 pre-manifest HD mutation carriers (36.3 ± 9 years, 42.1 ± 3.1 CAG repeats) and 14 controls (37.2 ± 10.3 years)	Decreased cerebral blood flow in medial and lateral prefrontal regions and increased in the precuneus and—in people close to symptom onset—in the putamen/increased in the hippocampus.	[142]
Human	11 people with manifest HD (42.1 ± 3.0 years, based on HD mutation and/or UHDRS); 9 controls (35.4 ± 3.2 years)	Blood flow velocity in the anterior cerebral artery was hyporeactive in people with HD during maze testing. This was linked more to motor planning and execution (maze tracing) than to problem thinking (maze solving).	[143]
Human	20 presymptomatic HD gene expansion carriers, (37.4 ± 9.1 years, 43.8 ± 2.4 CAG repeats), 24 controls (39.9 ± 8.7, <31 CAG repeats)	Reduced putamen volumes in HD and impaired basal ganglia perfusion.	[24]
Mouse	Male R6/2 HD model mice	Increasing impairments in NO-dependent vasodilation of the femoral artery in 12–16-week-old R6/2 animals, endothelial dysfunction due to impaired NO-dependent vasodilation in 16-week-old R6/2	[140]
Mouse	Male and female R6/1 HD model mice	NO levels were altered by dietary l-arginine (low: 0%, normal: 1.2%, or high: 5%), the dietary precursor of NO. High arginine increased cerebral blood flow and accelerated body weight loss and motor symptom onset. Low arginine reduced nitrotyrosine deposition and weight loss but not motor symptoms.	[141]

Notes. CAG, Cytosine, Adenine, Guanine—here abbreviated for CAG repeats; HD, Huntington’s disease; NO, nitric oxide.

## Data Availability

Not applicable.
